# Evaluation of short-term adverse events of COVID-19 vaccines: An observational study

**DOI:** 10.1097/MD.0000000000035549

**Published:** 2024-02-23

**Authors:** Fatemeh Fathi, Ali Ameri, Omid Safa, Mehdi Hassaniazad, Mohammad Fathalipour

**Affiliations:** a Student Research Committee, Faculty of Pharmacy, Hormozgan University of Medical Sciences, Bandar Abbas, Iran; b Department of Clinical Pharmacy, Faculty of Pharmacy, Hormozgan University of Medical Sciences, Bandar Abbas, Iran; c Infectious and Tropical Diseases Research Center, Hormozgan Health Institute, Hormozgan University of Medical Sciences, Bandar Abbas, Iran; d Department of Pharmacology and Toxicology, Faculty of Pharmacy, Hormozgan University of Medical Sciences, Bandar Abbas, Iran; e Department of Endocrinology and Metabolic Research Center, Hormozgan University of Medical Sciences, Bandar Abbas, Shiraz, Iran.

**Keywords:** Adverse events, AstraZeneca, COVID-19, Sinopharm, Sputnik V

## Abstract

Coronavirus disease 2019 (COVID-19) vaccines are the most effective tools in managing the pandemic. However, the concern about these vaccines is the occurrence of unwanted adverse events (AEs). This study aimed to evaluate the short-term AEs of COVID-19 vaccines (Sputnik V, Astrazenka, and Sinopharm). A cross-sectional study using an online questionnaire was conducted among 321 vaccinated individuals. Demographic information, history of drug use, prior infection with COVID-19, the type of vaccine, vaccination stage, local injection site complication, systemic complication, and allergic reactions were collected and evaluated. Local complications, including pain and swelling at the injection site, and systemic complications, including fever, fatigue, lethargy, lymphadenopathy, and diarrhea, were reported after the injection of the AstraZeneca vaccine was more than the other 2 vaccines; The prevalence of fatigue and lethargy was higher than other systemic complications. The least reported complication was due to lymphadenopathy. The Sinopharm vaccine showed a lower prevalence of AEs than the other 2. The rare AEs, such as facial paralysis, nasal bleeding, and urticarial, were further reported after injection of the AstraZeneca vaccine. In general, the severity of systemic complications after the second dose of the vaccine was also higher than the first dose. All 3 vaccines were safe and tolerable. The most commonly reported AEs were injection site pain (local) and fatigue and lethargy (systemic). These expected AEs occurred shortly after vaccination and indicated an early immune response after vaccination.

## 1. Introduction

The severe acute respiratory syndrome coronavirus 2 (SARS-CoV-2) is a new RNA virus from the coronavirus family that emerged from mutations in severe acute respiratory syndrome (SARS) and Middle East Respiratory Syndrome viruses with more severity and higher transmission speed as far as causing a pandemic called coronavirus disease 2019 (COVID-19).^[1]^ Unfortunately, no highly efficient treatment has been discovered for COVID-19 so far.^[2]^ The current way to prevent COVID-19 is prophylaxis by strengthening the immune system against SARS-CoV-2 by vaccination.^[3]^ Due to the high mortality caused by COVID-19 worldwide, it is necessary to carry out a global vaccination program.^[4]^ However, concern about the occurrence of unwanted adverse events (AEs) of these vaccines existed.

A previous study indicated that people who received the COVID-19 AstraZeneca vaccine rarely suffered from thrombotic adverse reactions.^[5]^ Another study conducted in Iraq and Jordan reported hypotension, shortness of breath, hyperglycemia, and chest pain rarely happened in people who received Pfizer, Sinopharm, and AstraZeneca vaccines. However, the local site reaction and the systemic AEs, such as fatigue, fever, headache, and body pain after receiving the first dose of Pfizer and AstraZeneca vaccines, significantly more happened than after receiving the first dose of the Sinopharm vaccine.^[6]^ The study had shown Sputnik V, AstraZeneca, and Sinopharm vaccines have AEs after first and secondary doses. However, the Sputnik V vaccine showed the most AEs and the Sinopharm vaccine showed the lowest AEs among these vaccines after the first and secondary doses. These vaccines’ most common AEs were fatigue, skeletal pain, and chill/fever.^[7]^ In another study, Bharat, Sputnik V, AstraZeneca, and Sinopharm vaccines had some adverse dermatological events such as urticarial and exanthematous rash in the injection site, angioedema, and eruption.^[8]^

Despite the studies conducted regarding the AEs of the designed vaccines, due to the prevalence of this vaccination, complete information about the AEs of vaccines (Sputnik V, Astrazenka, and Sinopharm) in different populations is still not available.^[9,10]^ Therefore, we tried to investigate the AEs of these vaccines that occurred shortly after receiving the vaccine.

## 2. Method

### 2.1. Study design

In this cross-sectional study, data collection about short-term AEs was carried out between August 1 and December 31, 2021. The participants in this study were people aged > 18 who had received at least the first dose or both doses of the vaccines while at least 72 hours had passed since the injection of the vaccine. This study was approved by the ethics committee of the Hormozgan University of Medical Sciences (IR.HUMS.REC.1400.096). A verbal informed consent was obtained from all participants.

### 2.2. Data collection

The information needed to conduct this study was collected through the creation of an electronic questionnaire. After the vaccination, the internet link of this questionnaire was sent to the vaccinated people so that the desired information of the study was registered in this system.

Collected data include:

- Demographic information includes age, gender, and history of underlying diseases (high blood pressure, diabetes, cardiovascular diseases, hyperlipidemia, multiple sclerosis, inflammatory bowel disease, chronic liver disease, chronic kidney disease, and chronic respiratory disease).
- History of allergy to a specific medicine or vaccine.
- History of taking drugs (corticosteroids, immunosuppressants, blood sugar reducers, blood pressure reducers, anticoagulants, oral contraceptive pills).
- The history of prior infection with COVID-19 and the date of infection was also recorded.
- Type of vaccine received, stage of vaccination, date of injection.
- Injection site complications (pain and swelling).
- Systemic AEs (fever, chills, headache, muscle pain, fatigue and lethargy, confusion, sore throat, lymphadenopathy, nausea and vomiting, diarrhea, gastrointestinal spasms, facial paralysis and swelling, allergic reactions including redness with itching, hives, shock).

### 2.3. Statistical analysis

The statistical analysis was conducted using SPSS V.18 (IBM Corp., Armonk, NY) with a significance level of *P* ≤ .05. The values reported for quantitative variables were mean ± standard deviation and for qualitative variables as frequency (percentage). The comparison of variables among the studied groups was made with the one-way ANOVA test for quantitative variables and the Chi-square test for qualitative variables.

## 3. Results

### 3.1. Population characteristics

In this study, a total of 321 vaccinated people were evaluated. Sixty people received the Sputnik V vaccine, 180 received the AstraZeneca vaccine, and 81 received the Sinopharm vaccine. The average age of the participants in the study is 35.75 years. The demographic and clinical characteristics of the participants in the study are demonstrated in (Table 1).

**Table 1:** Demographic and clinical characteristics of the participants.

Characteristics	All cases (n = 321)	Sputnik V (n = 60)	AstraZeneca (n = 180)	Sinopharm (n = 81)	*P* value
Age, yr	35.75 ± 10.95	40.10 ± 11.19	34.94 ± 8.69	34.32 ± 10.96	.003
18–35	184 (57.3)	24 (40.0)	109 (60.6)	51 (63.0)	.102
36–55	118 (36.8)	31 (51.7)	66 (36.7)	21 (25.9)	
>55	19 (5.9)	2 (8.3)	5 (2.8)	9 (11.1)	
Sex					
Male	107 (33.3)	19 (31.7)	62 (34.4)	26 (32.1)	.891
Female	214 (66.7)	41 (68.3)	118 (65.6)	55 (67.9)	
Underlying diseases					
HTN	19 (5.9)	6 (10.0)	10 (5.6)	3 (3.7)	.279
DM	9 (2.8)	2 (3.3)	3 (1.7)	4 (4.9)	.321
CVD	6 (1.9)	3 (0.5)	0 (0.0)	3 (3.7)	.017
Hyperlipidemia	10 (3.1)	2 (3.3)	2 (1.1)	6 (7.4)	.025
MS	1 (0.3)	0 (0.0)	1 (0.6)	0 (0.0)	.675
IBD	4 (1.2)	2 (3.3)	2 (1.1)	0 (0.0)	.205
CLD	3 (0.9)	0 (0.0)	3 (1.7)	0 (0.0)	.305
CKD	2 (0.6)	0 (0.0)	0 (0.0)	2 (2.5)	.076
CPD	3 (0.9)	0 (0.0)	3 (1.7)	0 (0.0)	.305
History of COVID-19	88 (27.4)	22 (36.7)	51 (28.3)	15 (18.5)	.053
History of allergy to medicines and vaccines	7 (2.2)	2 (3.3)	4 (2.2)	1 (1.2)	.699
Medication history					
Corticosteroids	9 (2.8)	1 (1.7)	5 (2.8)	3 (3.7)	.769
Immunosuppressants	5 (1.6)	2 (3.3)	3 (1.7)	0 (0.0)	.282
Antihyperglycemics	11 (3.4)	4 (6.7)	4 (2.2)	3 (3.7)	.258
Antihypertensies	21 (6.5)	7 (11.7)	10 (5.6)	4 (4.9)	.201
Anticoagulants	7 (2.2)	4 (6.7)	1 (0.6)	2 (2.5)	.019
OCPs	11 (3.4)	2 (3.3)	6 (3.3)	3 (3.7)	.988

Values were expressed as mean ± SD or median (interquartile range) for continuous variables and numbers (percentages) for categorical variables. Comparison between groups was performed with the One-way ANOVA test for continuous variables and the chi-square test for categorical variables.

CKD = chronic kidney disease, CLD = chronic liver disease, COVID-19 = Coronavirus disease 2019, CPD = chronic pulmonary disease, CVD = cardiovascular disease, DM = diabetes mellitus, HTN = hypertension, MS = multiple sclerosis, OCPs = Oral contraceptives pills.

### 3.2. Local AEs

In total, 267 local AEs were observed, including 231 cases of pain and 37 cases of inflammation at the injection site. The lowest number of pain at the injection site was related to the Sinopharm vaccine and inflammation at the injection site to the Sinopharm vaccine. The prevalence of local AEs of vaccines is indicated in (Table 2).

**Table 2:** Prevalence of solicited local and systemic AEs.

Adverse events	All cases (n = 321)	Sputnik V (n = 60)	AstraZeneca (n = 180)	Sinopharm (n = 81)	*P* value
Local					
Pain	231 (72.0)	47 (78.3)	150 (83.3)	34 (42.0)	.000
Swelling	37 (11.5)	10 (16.7)	25 (13.9)	2 (2.5)	.011
Systemic					
Fever	155 (48.3)	27 (45.0)	122 (67.8)	6 (7.4)	.000
Chill	123 (38.3)	19 (31.7)	101 (56.1)	3 (3.7)	.000
Headache	95 (29.6)	12 (20.0)	80 (44.4)	3 (3.7)	.000
Muscle pain	169 (52.6)	34 (56.7)	127 (70.6)	8 (9.9)	.000
Fatigue and malaise	188 (58.6)	37 (61.7)	138 (76.7)	13 (16.0)	.000
Confusion	82 (25.5)	15 (25.0)	63 (35.0)	4 (4.9)	.000
Sore throat	28 (8.7)	3 (5.0)	22 (12.2)	3 (3.7)	.041
Lymphadenopathy	8 (2.5)	0 (0.0)	8 (4.4)	0 (0.0)	.040
Nausea or vomiting	57 (17.8)	8 (13.3)	47 (26.1)	2 (2.5)	.000
Diarrhea	26 (8.1)	5 (8.3)	19 (10.6)	2 (2.5)	.086
Abdominal discomfort	40 (12.5)	5 (8.3)	33 (18.3)	2 (2.5)	.001
Rash and itching	9 (2.8)	2 (3.3)	7 (3.9)	0 (0.0)	.204

Values were expressed as numbers (percentages). Comparison between groups was performed with the chi-square test.

AEs = adverse events.

### 3.3. Systemic AEs

The observed systemic AEs included fever, chill, headache, muscle pain, fatigue and malaise, confusion, sore throat, lymphadenopathy, nausea or vomiting, diarrhea, abdominal discomfort, rash, and itching. The most common systemic AEs were fever, chills, muscle pain, fatigue, and malaise. Lymphadenopathy complication was observed only in the AstraZeneca vaccine. Rash and itching were not observed at all in the Sinopharm vaccine. The prevalence of systemic AEs is shown in (Table 2).

### 3.4. Unusual AEs

Generally, 20 cases of unusual AEs were observed, of which 12 cases were associated with the AstraZeneca vaccine, 5 cases with the Sputnik V vaccine, and 3 cases with the Sinopharm vaccine. These unusual AEs were observed, including facial paralysis, urticaria, diplopia, faintness, flushing, insomnia, nasal bleeding, nightmares, paresthesia, hair loss, and severe ageusia. The prevalence of unusual AEs is shown in (Table 3).

**Table 3:** Unusual symptoms reported by the participants.

Adverse events	Number of cases	Type of vaccine	Frequency (time) Duration (d)
Facial paralysis	3	AstraZeneca	One wk
Urticaria	1 2	Sputnik V AstraZeneca	
Diplopia	1	AstraZeneca	
Faint	1	Sputnik V	Two times
Flushing	2 1 1	Sputnik V AstraZeneca Sinopharm	
Insomnia	1 1	Sputnik V AstraZeneca	
Nasal bleeding	1	AstraZeneca	Two times
Nightmare	1	AstraZeneca	
Paresthesia	2	AstraZeneca	Four wk
Hair loss	1	Sinopharm	
Severe ageusia	1	Sinopharm	Two wk

Values were expressed as numbers.

### 3.5. First and second doses AEs

**Figure 1.**
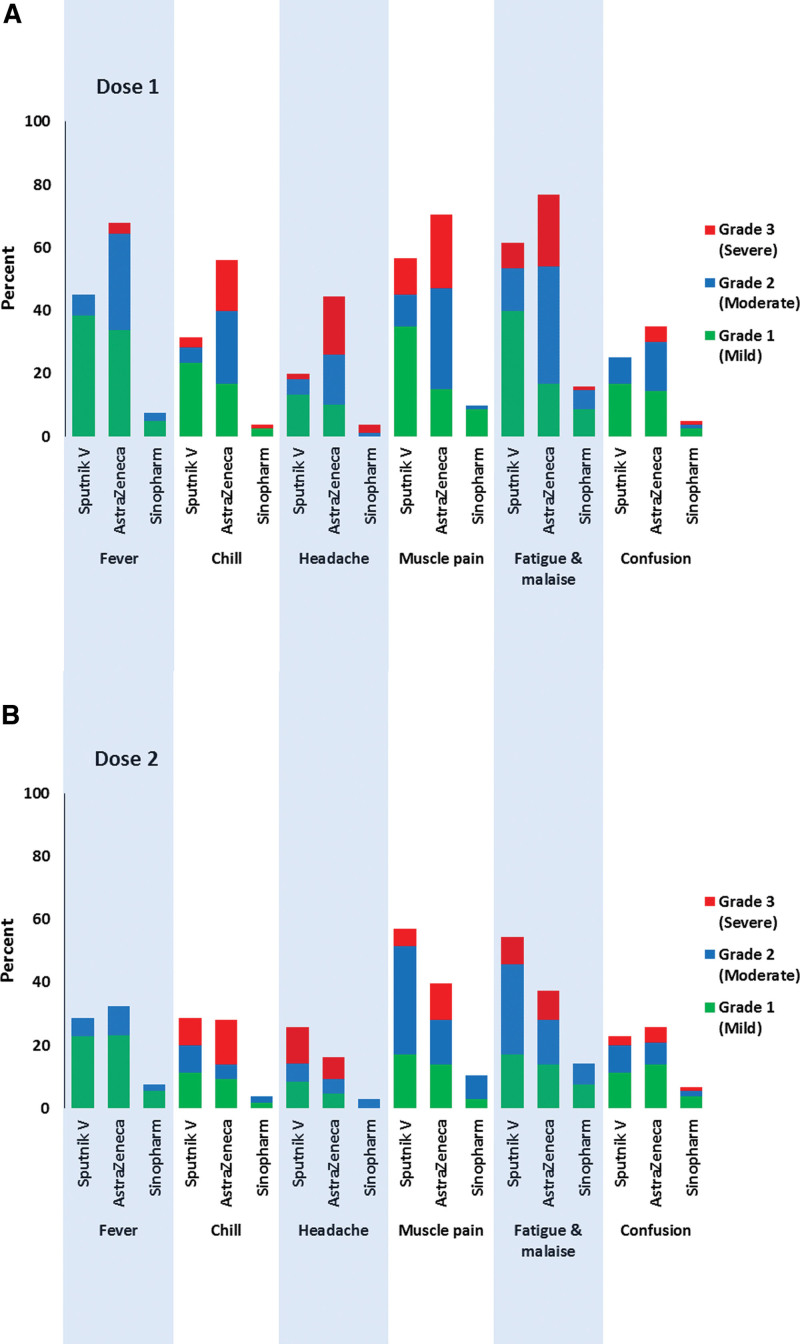
Prevalence of AEs stratified based on the doses. AEs = adverse events.

Fever, chills, headache, muscle pain, fatigue and lethargy, and confusion were AEs observed after both the first and second doses of vaccines. A comparison of the most common AEs and their severity according to the first and second doses of vaccines is shown in (Fig. 1).

## 4. Discussion

This cross-sectional study was conducted on COVID-19-vaccinated participants. We evaluated the local, systemic, and unusual AEs of the studied vaccines (Sputnik V, Astrazenka, and Sinopharm). Also, we compared the most common AEs and their severity between different vaccines and according to the first and second doses of these vaccines. Our study showed that among Sinopharm, AstraZeneca, and Sputnik V vaccines, the Sinopharm was the safest with the least AEs. The unusual AEs were rare in all these vaccines.

Our results indicated the most common systemic AEs include fatigue and lethargy, muscular pain, fever, chills, headache, and confusion, respectively. These results were similar to other studies; in a cross-sectional study conducted with 4755 participants, the AEs of common vaccines used in Iran were investigated. The most common complications reported included fatigue and lethargy, fever and chills, and muscle pain.^[7]^ In a cross-sectional descriptive study based on a questionnaire, the potential AEs of the COVID-19 vaccines (Astrazenka, Sputnik V, Sinopharm, and Bharat) were investigated. Skin reactions occurred in 30% of people vaccinated against COVID-19, and the most common skin reactions were local reactions at the injection site, exanthematous rash, and urticarial.^[8]^ In another study, which was based on a questionnaire among 1205 dental students and dentists in Iran, to investigate the AEs of Sinopharm, Sputnik V, AstraZeneca, and Covexin vaccines, the most common AEs reported in this study included fatigue, pain at the injection site, and lethargy.^[11]^

Our study reported the AEs of the AstraZeneca vaccine were generally higher than Sinopharm, and Sputnik V and Sinopharm vaccines had the least AEs among these vaccines. This result can be consistent with or contradict other studies; For example, the cross-sectional study conducted in Iran with 4755 participants. The least AEs were associated with the Sinopharm vaccine, the same as our study. Still, they reported the Sputnik V vaccine had a higher rate of symptoms than those who received the AstraZeneca vaccine.^[7]^ On the other hand, in a study involving 1440 and 80 healthcare personnel who received a single dose of the Pfizer and AstraZeneca vaccines, respectively, more severe symptoms were reported associated with the administration of the AstraZeneca vaccine.^[12]^ Based on the findings of Montalti et al study, the severity of local and systemic AEs after the second dose of his Sputnik vaccine was higher than the first dose. In the results of this study, the number of people who experienced severe AEs from this vaccine increased in the second dose compared to the first dose. Still, it is noteworthy that the fever complication in both doses of this vaccine lasts up to 48 hours. The number of people who reported fever was also lower in the second dose.^[13]^

The lowest AEs reported in our study, both in the first dose and in the second dose, were observed with the Sinopharm vaccine, which was consistent with previous studies; For example, in a double-blind, randomized, placebo-controlled clinical trial conducted in Henan Province, China, the mean cumulative AEs recorded after 7 days of Sinopharm vaccine administration in phases 1 and 2 of the study were 15%. The most commonly reported AEs were injection site pain and fever.^[14]^

Our research had shown that the most common AEs reported after receiving the AstraZeneca vaccine were pain at the injection site with a prevalence of 83.3%, fatigue, lethargy (76.7%), and muscle pain (70.6%), fever (67.8%), and chills (56.1%), which is consistent with other studies. In a study that aimed to describe the AEs of COVID-19 vaccines in Jordan, 1086 people participated in this study, of which 358 people received the AstraZeneca vaccine; The most common AEs recorded for this vaccine were injection site pain (83.8%), fatigue (76.5%), muscle pain (66.2%), fever (56.7%), chills (61.5%) and headache (51.4%).^[12]^ Three hundred eleven people participated in a study in Bahrain to investigate the AEs of COVID-19 vaccines, and the most common AEs reported after receiving the AstraZeneca vaccine were pain at the injection site (31.03%), fever (27.6%) and fatigue (10.35%).^[15]^

In the current study, 3 cases of facial paralysis were reported, and all 3 were reported with the AstraZeneca vaccine. According to the report of the Medicines and Healthcare Products Regulatory Agency, until December 15, 2021, among all recipients of the AstraZeneca vaccine, 608 cases of facial paralysis were reported after receiving the vaccine.^[16]^

One case of hair loss was reported 2 weeks after receiving the Sinopharm vaccine in our study. This complication has also been reported with other vaccines; For example, in 1 study, 3 cases of hair loss were reported (one case after receiving the Pfizer-Biotech vaccine and 2 cases after receiving the AstraZeneca vaccine), and all 3 had a history of hair loss before receiving the COVID-19 vaccine. In people with dysregulated inflammatory pathways, interactions between the immune system and vaccines may enhance autoimmune mechanisms. In these cases, the vaccine may focus on components that play a crucial role in both the COVID-19 vaccination and the pathogenesis of hair loss by causing hair loss.^[17]^

In our study, no case of anaphylaxis was reported. The only allergic reactions that were recorded in this study were rash and itching with a prevalence of 9 cases out of 321 (2.8%), 2 of which were Sputnik V vaccine, and 7 of them had received the AstraZeneca vaccine; Also, 1 person who received Sputnik V vaccine and 2 people who received AstraZeneca vaccine reported hives. In a cross-sectional study, 1 case of urticaria and 2 cases of hot flashes were reported after receiving the Sputnik vaccine without recording the duration of AEs.^[18]^ Studies have shown that mRNA-based vaccines can cause allergic reactions and anaphylaxis.^[19]^ One of the materials used as an adjuvant in vaccines is polyethylene glycol (PEG). The presence of PEG in the vaccine can cause allergic reactions and anaphylaxis. The lower the molecular weight of PEG, the less the possibility of allergic reactions.^[20]^

This study had several limitations: Most reported symptoms occurred in the initial stage after vaccination, and the mid-term and long-term effects of vaccines were not included. The non-cooperation of the participants in completing the questionnaire wholly and correctly caused a decrease in the size of the statistical sample and the accuracy of the results. Due to the small number of analysis data in this study, according to the consultant professor and statistical expert of the study, it is possible to investigate the relationship between the incidence and severity of the short-term AEs of the COVID-19 vaccines with the age and gender of the people, history of infection. There was no COVID-19 disease, underlying diseases of the people, and the drugs used by the vaccinated people. Some of the AEs of vaccines may closely resemble the symptoms of people underlying conditions. After experiencing this symptom, the person may not be able to decide whether this experimental symptom was associated with the vaccine AEs or the disease symptoms. Be distinguished based on context.

## 5. Conclusion

The observed signs and symptoms show that all 3 vaccines are safe and tolerable. The Sinopharm vaccine showed a lower incidence of AEs than the other 2 vaccines. The most common AEs included local reactions at the injection site and systemic events of fatigue and lethargy, muscle pain, and fever. These typical and expected AEs occurred shortly after vaccination and indicated the initial reaction of the immune system after vaccination. In general, the AEs caused by vaccines should be carefully evaluated in prospective clinical trial studies with larger sample sizes.

## Acknowledgments

We appreciate and thank all participants, whose help and participation made this study possible.

## Author contributions

**Conceptualization:** Fatemeh Fathi, Ali Ameri, Mohammad Fathalipour.

**Data curation:** Ali Ameri, Mohammad Fathalipour.

**Investigation:** Fatemeh Fathi, Omid Safa, Mehdi Hassaniazad.

**Methodology:** Fatemeh Fathi, Ali Ameri, Mohammad Fathalipour.

**Software:** Fatemeh Fathi, Ali Ameri, Mohammad Fathalipour.

**Supervision:** Fatemeh Fathi, Ali Ameri.

**Validation:** Mohammad Fathalipour.

**Visualization:** Fatemeh Fathi, Omid Safa, Mehdi Hassaniazad.

**Writing – original draft:** Ali Ameri, Mohammad Fathalipour.

**Writing – review & editing:** Mohammad Fathalipour.
